# Total rhinectomy for nasal carcinomas^☆^

**DOI:** 10.1016/j.bjorl.2019.06.002

**Published:** 2019-07-02

**Authors:** Fábio Muradás Girardi, Luiz Alberto Hauth, Aliende Lengler Abentroth

**Affiliations:** Hospital Ana Nery, Departamento de Cirurgia de Cabeça e Pescoço, Santa Cruz do Sul, RS, Brazil

**Keywords:** Carcinoma, squamous cell, Lymphatic metastasis, Nose neoplasms, Skin neoplasms, Survival, Carcinoma espinocelular, Metástase linfática, Neoplasias nasais, Neoplasias cutâneas, Sobrevida

## Abstract

**Introduction:**

Total rhinectomy is an uncommon procedure for the treatment of nasal malignancies, usually reserved for locally advanced tumors. There are few case series studying total rhinectomy in the literature, reporting conflicting results about recurrence and metastasis.

**Objective:**

Evaluate prognosis of total rhinectomy cases for malignant neoplasia in our institution.

**Methods:**

Retrospective review from January 2013 to September 2018, including all patients undergoing total rhinectomy in our Institution, under the care of the Head and Neck surgical team.

**Results:**

Ten patients were included, two men and eight women. The mean patient age was 71.6 years old. The majority had nasal skin (8 cases) carcinomas. Squamous cell carcinoma was present in seven cases. In total, six cases had regional metastasis, in a median period of 14.3 months. The overall mortality and disease specific mortality was 50% and 30%, respectively, in a median follow-up of 45.7 months.

**Conclusion:**

We observed high overall and disease-specific mortality among cases with advanced nasal malignancies undergoing total rhinectomy.

## Resumo

**Introdução:**

A rinectomia total é um procedimento incomum para o tratamento de neoplasias nasais, geralmente reservado para tumores localmente avançados. Há poucas séries de casos que estudam a rinectomia total na literatura, as quais descrevem resultados conflitantes sobre recorrência e metástase.

**Objetivo:**

Avaliar o prognóstico de pacientes submetidos a rinectomia total por neoplasia maligna em nossa instituição.

**Método:**

Revisão retrospectiva de janeiro de 2013 a setembro de 2018, incluiu todos os pacientes submetidos a rinectomia total em nossa instituição, sob os cuidados da equipe de cirurgia de cabeça e pescoço.

**Resultados:**

Dez pacientes foram incluídos, dois homens e oito mulheres. A média de idade dos pacientes foi de 71,6 anos. A maioria apresentava carcinoma da pele nasal (oito casos). O carcinoma espinocelular estava presente em sete casos. Seis casos tiveram metástase regional em um período mediano de 14,3 meses. A mortalidade geral e a mortalidade específica da doença foram de 50% e 30%, respectivamente; o acompanhamento médio foi de 45,7 meses.

**Conclusão:**

Observamos alta mortalidade geral e específica da doença entre os casos com neoplasias nasais avançadas submetidas à rinectomia total.

## Introduction

Skin cancer is the most common head and neck malignant neoplasia in the vast majority of the world. The nose is a typical subsite of head and neck skin cancers, reaching almost 50% of them in some series^1^ (15% in our series — unpublished data). The majority of malignancies are diagnosed in earlier stages, requiring limited excisions, many times performed by different specialists. Total rhinectomy (TR) is an exception procedure, reserved for locally advanced tumors. There are few case series studying TR in the literature,^2^, ^3^, ^4^, ^5^ most of them grouping different histologies, and combining mucosal and skin tumors.

About 75–80% of nasal skin malignant tumors are Basal Cell Carcinomas (BCC), similar to other head and neck topographies.^1^ Squamous Cell Carcinomas (SCC) comprise the other 20–25%. Although central face topography is considered among NCCN high risk features for cutaneous SCC of head and neck,^6^ regional metastasis are usually associated with large size, deeply invasive lesions and high grade tumors, often with perineural and lymphovascular invasion.^7^

Our Head and Neck Department is the reference center for skin cancer cases. Many of our patients are of German descent, a significant part of them linked to agricultural work.

The purpose of this study was to evaluate diagnosis, treatment, and outcomes in a case series of TR for nasal carcinomas.

## Methods

A local institutional review board and a regional Research Ethics Committee approved the study protocol (CAAE: 93792318.4.0000.5304). We undertook a retrospective review from January 2013 to September 2018, including all patients undergoing TR in our Institution, under the care of the Head and Neck surgical team. The cases were identified from operating theatre records and consultant diaries. Case notes were reviewed to gather information relating to clinical features, histopathological reports, surgical treatment and outcomes. Tumors were classified according to the TNM classification system of the 8th edition of the American Joint Committee on Cancer.

## Results

**Figure 1:**
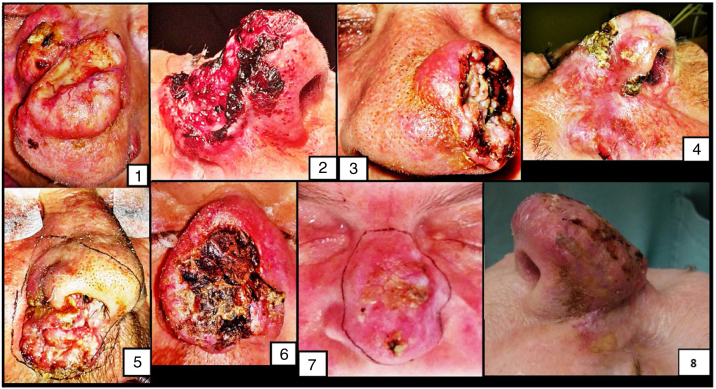
Clinical images of the first eight cases submitted to total rhinectomy.

During the analyzed period, 10 patients, two men and eight women were underwent TR due to malignant neoplasia of the nose at our tertiary cancer care center (Fig. 1). Only one was a immunosuppressed patient. The mean patient's age was 71.6 years old (range 56.4–87.2 yo). Pathological information is summarized in Table 1. In 7 cases there were treatment attempts prior to TR, five of them treated with surgical resections with compromised surgical margins, and the two remaining cases, with liquid nitrogen therapy for several times. The most frequent tumor location was the nasal skin (8 cases). The nasal vestibule was the tumor epicenter in the two remaining cases. The nasal dorsum was the most common skin subsite (5 cases). Histological analysis of specimens revealed a diagnosis of BCC in three patients and SCC in seven patients In all cases, ulcerated lesions existed. The mean tumor diameter was 3.6 cm (range 1.5–6 cm). All cases were classified as Clark V, and in three of them, there was bone invasion. In one case, final histology showed compromised surgical margins. In six cases there was vascular or perineural invasion. Two cases with SCC had clinically suspicious neck lymph nodes at the first consultation in our department, but the other four SCC cases recurred in the neck or parotids in a median period of 14.3 months (range 2.3–28.9 months). In total, three cases showed extracapsular spread. Except for the only case with SCC without regional metastasis after 18.5 months of follow-up, all other patients with SCC received adjuvant radiotherapy, two of them associated with chemotherapy. In three patients, adjuvant treatment was definitely interrupted by critical side effects. Since in our department we do not have access to maxillofacial prosthetic rehabilitation, the majority (9 cases) was reconstructed with surgical flaps. The overall mortality was 50%. The median follow-up was 45.7 months (range 18.5–66.1 months). All deaths were among SCC cases with regional metastasis, although in only three cases the deaths were directly associated to the disease.

**Table 1:** Clinical and pathological information from TR cases.

	Age	Sex	Histology	Diameter	DOI	TNM		Recurrence	Follow	OS	DRD
Case 1	87.2	F	SCC	4.5	NI	pT4aNxM0	IV	No	27.0	Alive	No
Case 2	77.3	F	BCC	6.0	NI	pT4aNxM0	IV	No	36.2	Alive	No
Case 3	63.8	F	SCC	3.0	NI	pT3N1M0	III	Yes	15.3	Dead	Yes
Case 4	80.3	F	BCC	2.8	NI	pT3NxM0	III	Yes	29.0	Dead	No
Case 5	75.7	F	SCC	3.0	2.7	pT3NxM0	III	Yes	15.2	Dead	No
Case 6	59.3	M	SCC	4.0	2.0	pT3NxM0	III	No	18.5	Alive	No
Case 7	67.0	F	SCC	3.3	1.3	pT3NxM0	III	Yes	48.5	Alive	No
Case 8	74.0	F	SCC	4.4	1.8	pT3NxM0	III	Yes	5.8	Dead	Yes
Case 9	74.8	F	SCC	3.5	NI	pT4aNxM0	IV	Yes	26.0	Dead	Yes
Case 10	56.4	M	BCC	1.5	0.8	pT3NxM0	III	No	66.1	Alive	No

M, male; F, female; SCC, squamous cell carcinoma; BCC, basal cell carcinoma; DOI, depth of invasion; diameter and DOI are expressed in cm; TNM, 8th edition of the American Joint Committee on Cancer (at first presentation); follow is expressed in months; OS, overall survival; DRD, disease related death.

## Discussion

We described an institutional series about TR for advanced cutaneous or vestibular carcinomas. Most of our cases were skin SCC. Often the procedure was an “end-stage” therapy, done frequently after multiple prior surgical attempts at tumor ablation. Although there are encouraging results in the literature for primary definitive radiotherapy in nasal malignancies, severe side effects were also reported,^8^ and long-term control is generally best achieved with surgical standard approaches.^9^

Few case series have been published about this issue. Our results show that cases with advanced nasal carcinomas, especially SCC, display aggressive behavior, with a high incidence of regional metastasis, similar to other reports of advanced skin SCC from other head and neck subsites^10^, ^11^ or in midfacial location.^12^ Despite high prevalence of regional disease, most authors recommend conservative measures regarding the cervical region, even in advanced cases.^12^ Disease-related mortality seems to be associated with regional metastasis and/or recurrence. Overall mortality may be high, especially in series like ours, with high mean age.

Stanley described the greatest total rhinectomy series in 1988: fifty-one cases, most of them middle-aged patients, 25 of them with SCC comprising the larger lesions. As found in other series, more than a half of them had previous unsuccessful surgical attempts to cure their disease, many of them undergoing multiple previous excisions. The authors described an overall survival of 50% and a 21% disease related mortality in a mean follow-up of 68 months, similar to our results. Recurrence was observed in half of the patients and was apparently related to worse survival, as about 50% of recurrent tumor patients died of their disease.^4^

Conflicting results were published by Harrison in 1982. In his personal experience in TR over 15 years, most of them with septal SCC, he reported none with nodal metastasis. Local recurrence and uncontrolled disease were the main follow-up problems.^3^ For Becker et al., analyzing a single institution experience with nasal cavity SCC, regional metastasis were also rare at initial clinical presentation, with only on case. In this case series, 14 patients were treated with TR.^9^ Subramaniam et al. also reported a series of nine cases without any disease -related death in a median follow-up of 5 years (although three cases had less than one year of follow-up).^5^

Unfortunately facial prosthesis rehabilitation is not routinely available for public health system- assisted patients in our country. Therefore, the majority of our cases was submitted to surgical reconstruction. As observed by Stanley et al.^4^ TR is not a technically difficult procedure. Nevertheless, patient rehabilitation is a real problem. Even in the best hands, using new techniques and multiple interventions, for many patients, a complete nasal reconstruction often only succeeds in making a horrible situation look strange.^4^

## Conclusion

We observed high loco-regional uncontrolled disease and high overall and disease specific mortality among cases with advanced nasal skin and vestibular malignancies, especially SCC.

## Conflicts of interest

The authors declare no conflicts of interest.
